# Central nervous system biomarkers for antiobesity drug development^☆^

**DOI:** 10.1016/j.drudis.2013.08.015

**Published:** 2013-12

**Authors:** Hisham Ziauddeen, Paul C. Fletcher

**Affiliations:** 1 Department of Psychiatry, Behavioural & Clinical Neuroscience Institute, Cambridge Biomedical Campus, University of Cambridge, UK; 2 Wellcome Trust MRC Metabolic Research Laboratories, Institute of Metabolic Science, University of Cambridge, Cambridge, UK; 3 Cambridgeshire & Peterborough NHS Foundation Trust (CPFT), Cambridge, UK

## Abstract

With antiobesity agents, weight loss can emerge from an array of metabolic, cognitive and behavioural changes that translate into weight change over time. In early drug development, characterising these changes can actually be more informative than simply measuring weight loss. Biomarkers for these mechanisms can be used to determine whether potential compounds are worth developing further by providing proof of mechanistic action and detecting early signs of neuropsychiatric adverse effects. In this review, we examine potential biomarkers for effects on metabolism and satiety, hedonics and motivation, and eating behaviour. We also review biomarkers for early detection of neuropsychiatric adverse effects.

## Highlights

• Neuroimaging, cognitive and behavioural biomarkers can aid antiobesity drug development.
• These biomarkers can detect early signals of mechanistic efficacy and adverse effects.
• In Phase II biomarkers can provide proof-of-concept to inform the decision to advance to Phase III.
• Potential biomarker candidates that have been used with antiobesity drugs are discussed.
• These candidate biomarkers need further exploration, standardisation and validation.

## Introduction

During the past five years, several antiobesity agents have been withdrawn at different stages in the drug development process because of concerns about limited efficacy, safety or both. The most notable have been rimonbant, withdrawn because of its neuropsychiatric adverse effects (depression, suicidality) [1], and sibutramine, because of its cardiovascular risks [2]. 2012 saw the first FDA approvals for two antiobesity agents since orlistat in 1999. These were lorcaserin (selective serotonin 5-HT2C agonist) [3] and Qysmia^®^ (the stimulant phentermine, which suppresses appetite and increases metabolic rate, and the antiepileptic topiramate) [4]. However, neither was approved by the European Medicines Agency (EMEA), which was not sufficiently satisfied about the safety of either drug [5,6]. Contrave^®^ (noradrenaline and dopamine re-uptake inhibitor buproprion and mu-opioid antagonist naltrexone) received a positive recommendation from the FDA advisory committee in late 2010 but was not approved because of safety concerns [7,8]. It is currently in further Phase III trials. Another combination Empatic™ (buproprion and the antiepileptic zonisamide) has completed Phase IIb [9] and both these agents show promising early efficacy signals. However, over this period several other agents have not progressed. The D3-antagonist GSK588089 failed to demonstrate mechanistic efficacy in Phase I [10,11]. The cannabinoid CB1 receptor inverse agonist taranabant [12,13] and antagonist otenabant [14] showed similar adverse effects to rimonabant. The development of metreleptin/pramlinitide (leptin analogue and synthetic amylin) was terminated possibly following antibody reactions to metreleptin [15]. Agents currently in Phase I and II include the mu-opioid receptor (MOR) antagonist GSK1521498, the triple monoamine reuptake inhibitor tesofensine [16,17], the glucagon-like peptide 1 (GLP-1) analogue liraglutide [18], the neuropeptide Y (Y1 and Y5) agonist velneperit, the neutral cannabinoid antagonists and the MC4R receptor agonist RM493 [19].

This is not a comprehensive summary of the state of the field and is only meant to highlight the challenges for, and the very modest returns from, a large and costly antiobesity drug development programme. The challenge is partly due to the licensing regulatory requirement(s) of the FDA and the EMEA. Both require an agent to demonstrate statistically significant weight loss (compared with placebo) at one year in large Phase III trials [20,21]. The FDA also requires combination treatments to demonstrate superiority over each individual component before Phase III. An acceptable safety profile is crucial and neuropsychiatric adverse effects and abuse liability are particular concerns with centrally acting agents. Additionally, in 2012 the FDA advisory committee recommended that all antiobesity drugs should demonstrate cardiovascular safety (regardless of theoretical risk or signal of cardiovascular harm) before approval [22]. The journey to licensing for a new agent is, therefore, necessarily a long and costly one.

In this review we consider whether biomarkers can be used to provide early signals of efficacy and/or adverse effects in Phase I and II to enable early go–no-go decisions and possibly have value as surrogate endpoints. Our focus here is on neuroimaging and neurobehavioural measures for centrally acting agents. There are very few examples of this experimental medicine approach in obesity with fewer replications and no real validation studies of these biomarkers. Our aim therefore is to present some potential candidates for development and validation. We also discuss their limitations and, particularly, the question of what biomarkers add to the body weight signal that is ultimately paramount for efficacy and regulatory approval.

## Some difficulties in antiobesity drug development

We would first briefly like to highlight three potential problems in the field that have been elegantly discussed previously [23]. The first is that the weight loss endpoint is some way downstream of the target mechanism of a dug and can result from a combination of different mechanisms acting simultaneously or successively. Given the complex multiple pathways to positive energy balance and consequent obesity [24], it seems unlikely that it would be possible to target safely even a very proximal pathway such as orexigenic drive in the hypothalamus to a sufficient extent to produce significant and, crucially, sustained weight loss, without addressing the processes distal to this. The second is that to focus on weight loss is to focus on the ‘consequences rather than the causes’ [23]. The mechanisms that led to the initial weight gain need to be treated alongside or after weight loss treatment given the high risk of weight regain. There could be considerable value in drugs that facilitate weight maintenance by targeting these causative mechanisms, even if they do not achieve weight loss. A third difficulty is the conceptualisation of obesity as a homogenous syndrome that should respond predictably to an antiobesity treatment. It is likely that common obesity is a very heterogeneous syndrome, even without accounting for comorbidities (e.g. Type 2 diabetes). Although diet and lifestyle modification are invaluable as general treatments and at the public health level, treating a specific patient might require a more individualised approach. It could also be the case that certain eating behaviours (e.g. binge eating) and pharmacogenotypes (e.g. MOR *OPRM1* gene A118G polymorphism) [25] are more amenable to specific treatments. This might not be gleaned from heterogenous Phase III trial populations that have not been stratified *a priori* to permit the necessary subgroup analyses and makes a case for defining a target subgroup in Phase II, based on the predicted mechanism of action of the agent. This does however present theoretical and practical challenges in terms of defining and recruiting the relevant subgroup.

For the purposes of this article, we take the view of obesity as a heterogenous syndrome with a multifactorial causation requiring a multipronged and individualised treatment, of which pharmacology would be one prong. From this perspective we would argue that the first requirement for a potential antiobesity agent is that it should reliably and safely affect a specific target mechanism or behaviour and it is here that we think that biomarkers could be very valuable.

## The value of biomarkers in early drug development

In drug development, a biomarker is a characteristic that is objectively measured and evaluated as an indicator of pharmacological response to a treatment. They can be used to monitor or predict treatment response and can even serve as surrogate endpoints [26].

What would be the value of biomarkers in antiobesity drug development? First, they could offer clear evidence of mechanistic efficacy and could permit the detection of signals that would not be immediately available (e.g. shifting food preference and desire, subtle changes in mood) or be less easily accessible from subjective report (e.g. attention bias to food or altered emotional processing). Second, they can aid dose optimisation and the examination of synergistic effects of combination treatments. Third, if a drug has a proven mechanistic effect yet fails to cause sufficient weight loss after optimal dosing it would suggest that modulating that mechanism alone is insufficient for weight loss. Fourth, they could aid in the early identification of adverse effects. Fifth, they could also enable identification of target subgroups. These particular uses would help decisions about proceeding with development and optimising the process. Finally, a biomarker that predicts efficacy would be an invaluable surrogate endpoint in the drug development process. We will now examine some potential biomarkers and their applications, present and potential (Table 1).

## Biomarkers for mechanisms

### Functional neuroimaging

**Figure 1:**
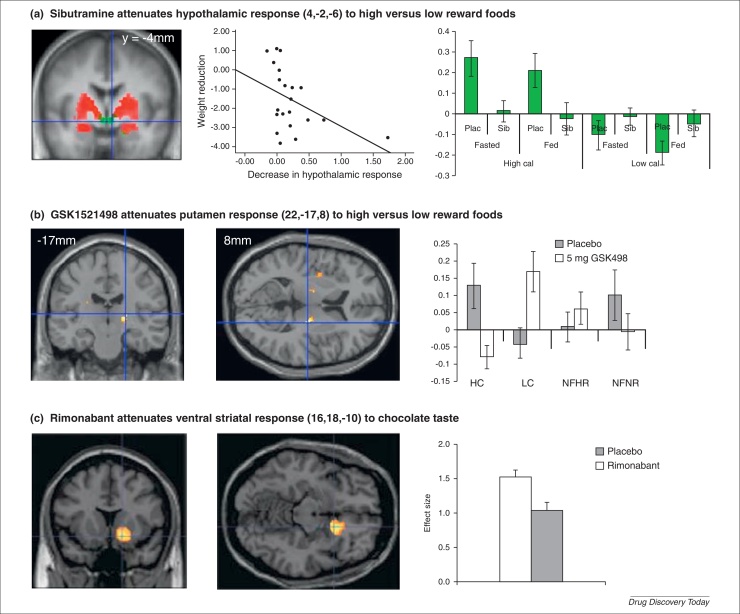
Neuroimaging biomarkers. **(a)** Shows the attenuation of neural activation in the hypothalamus (coordinates 4, −2, −6) by sibutramine (*P* = 0.014, small volume and Bonferroni corrected) on the left [28]. This attenuation was correlated with the reduction in weight in the 11 subjects who received placebo first in the crossover (Spearman's rho = −0.81, *P* = 0.03). The parameter estimates from this region shown on the right demonstrates the specific effect of sibutramine in response to rewarding foods (high cal) independent of fed/fasted state. **(b)** Shows the attenuation of the response in the putamen (22, −17, 8) to rewarding foods produced by GSK1521498 [*P* < 0.05, Family Wise error (FWE) corrected]. The parameter estimates on the right show that this effect is specific to food and is produced by an attenuation of the response to rewarding foods (HF) and an enhanced response to less rewarding foods (LF) [37]. **(c)** Displays the attenuating effect of rimonabant on the ventral striatal response (16, 18, −10) to the taste of chocolate in the mouth (*P* < 0.05, FWE corrected). Parameter estimates are shown on the right [68].

At present, functional magnetic resonance imaging (fMRI), in the context of a relevant cognitive task or state, remains the best option for examining the neural effects of a drug. A useful fMRI task for evaluating antiobesity drugs is viewing pictures of foods. It is associated with robust neural responses (enhanced blood flow) in key areas of the reward circuit (e.g. ventral striatum, orbitofrontal cortex and amygdala) and the homeostatic system (e.g. hypothalamus) when viewing rewarding foods compared to less rewarding foods, or foods compared to non-foods [27–30]. These responses are enhanced by hunger and attenuated by satiety [31–34]. A drug that decreases appetite would be predicted to attenuate the expected enhancement produced by the fasted state and one that enhances satiety to enhance the attenuation produced by the sated state. In overweight subjects, sibutramine 15 mg/day for two weeks attenuated the hypothalamic and amygdala responses to rewarding foods compared with less rewarding foods, independent of whether participants were fed or fasted [28] (Fig. 1a). This suggests that sibutramine reciprocally increases the anorexigenic drive from hypothalamic pro-opiomelanocortin (POMC) neurons and decreases the orexigenic drive from Agouti-related protein (AgRP) neurons [35], as would be predicted by its serotonergic action. Further, the degree of hypothalamic suppression correlated with *ad libitum* intake and weight loss during the study. However, in a study in obese subjects using the same treatment regime but a different analysis approach, sibutramine enhanced amygdala activity when viewing rewarding foods compared with non-foods [36]. This difference emphasises a particularly important point with respect to the use of functional neuroimaging as a biomarker: it produces measures that are highly stimulus- and context-specific as well as sensitive to different data analysis strategies. Thus, if the measures are to be useful for studying other agents targeting appetite or satiety (e.g. lorcaserin, velneperit, liraglutide and tesofensine) then it would be important to develop standardised and comparable approaches across studies.

A drug that attenuates the reward value of food would be predicted to cause a corresponding attenuation of reward circuit activity. In obese subjects 4 weeks of treatment with MOR antagonist GSK1521498 produced a specific attenuation in a region of the putamen in response to viewing rewarding compared with less rewarding food pictures [37] (Fig. 1b). Although the spatial resolution of fMRI does not permit us definitely to say so, this particular region might be the ventral pallidum, the site of a ‘hedonic hotspot’ [38], rich in MORs, and a target region for an agent that modulates the hedonics of food reward. Interestingly, subjective liking ratings for the food images were unchanged. This task can also be adapted to examine the specificity of effects to food by including a category of rewarding non-foods as was done in this study and the drug effect was found to be specific to food images only [37]. Given the animal data supporting the role of D3-receptors in drug and food seeking [39], GSK598809 was developed for the treatment of compulsive overeating and obesity. However, single doses of GSK598808 175 mg in overweight binge eaters had no effect on this task [11].fMRI can also be used to study the neural responses to food consumption by delivering liquid rewards to the subject in the scanner. In healthy normal-weight volunteers, rimonabant 20 mg for 7 days attenuated ventral striatal and putamen responses to the taste of chocolate milk (Fig. 1c). Subjects reported no subjective change in mood or liking for chocolate, but did report a decrease in chocolate intake during the treatment period [40]. The study also reported enhanced lateral orbitofrontal cortex (OFC) response to an aversive liquid. Once again with a hedonic system modulator we see a very early signal of a mechanistic effect. However, this study was terminated prematurely by the withdrawal of rimonabant and no further examination of these effects or their specificity to food have been reported.

The food pictures and the liquid reward delivery tasks would be useful for evaluating other agents targeting hedonic systems (e.g. the neutral and the peripherally restricted cannabinoid antagonists) [41]. The neutral antagonists lack the inverse agonism of rimonabant and taranabant, and are less likely to disrupt constitutive endocannabinoid signalling, potentially posing less neuropsychiatric risk [42]. However it would be important to examine the specificity of their effects. The peripherally restricted antagonists are hypothesised to affect cannabinoid-mediated lipogenesis and appetite signalling in the periphery [43,44]. Here the important question is whether these agents produce the same effects as their centrally acting counterparts (absence of a direct central action does not exclude secondary effects of peripheral antagonism on neural activation). Of course, positron emission tomography (PET) imaging would be the technique for assessment of central nervous system (CNS) penetration of the peripherally restricted agents [45].

The food pictures task has also been demonstrated to be sensitive to the effect of gut hormones. Administration of leptin in leptin-deficient patients restores the normal hunger and satiety neural responses [46]. Ghrelin increases responses to food images in the amygdala, OFC and striatum [47]. There is also preliminary evidence that Peptide YY (PYY) and GLP-1 attenuate activity in the amygdala, OFC, caudate, accumbens and insula, when viewing high calorie food pictures in the fasted state [48]. These findings are relevant to the development of potential agents based on these gut hormones.

Before we conclude this section it is important to acknowledge the tremendous variability in the findings in the fMRI literature using this task [49]. This is partly related to the lack of standardisation of this task or the analysis strategy but there is also genuine individual variability. This is a major limitation when it comes to comparing studies and developing biomarkers. At least at present, using fMRI measures meaningfully requires a pre-treatment baseline scan followed by a treatment scan to examine changes from the baseline.

### Cognition and behaviour

Assays of cognition and behaviour offer ways of identifying subtle changes, even those inaccessible to consciousness that could herald a key therapeutic effect when subjective measures and overt changes are not (yet) manifest.

#### Attention bias measures

**Figure 2:**
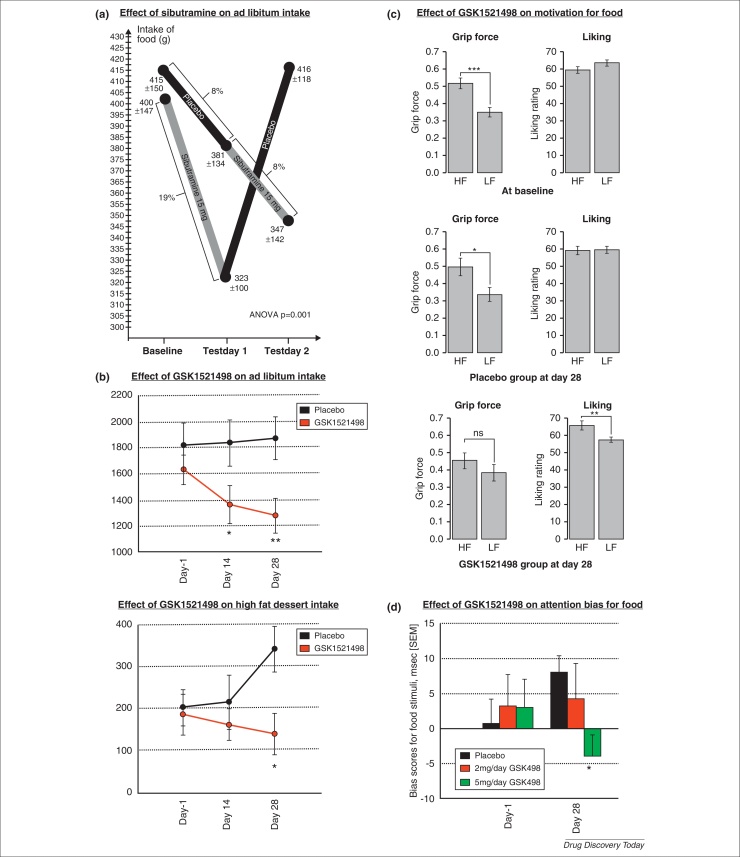
Behavioural biomarkers. **(a)** Effect of 14 days of sibutramine on intake in a placebo crossover design, sibutramine decreases intake, which recovers on placebo [54]. **(b)** Effect of 28 days of GSK1521498 on intake on an *ad libitum* buffet. The second graph shows a significant effect on high fat desserts (*P < 0.05) [25]. **(c)** Effect of 28 days of GSK1521498 on grip-force exerted to view high fat food images (HF) compared to low fat images (LF). Greater effort is exerted to view HF images at baseline but this attenuated in the treatment group but persists in the treatment group. Liking ratings also discriminate between HF and LF images in the treatment group alone [37]. **(d)** GSK1521498 5 mg/day for 28 days reduces the attentional bias for food [51].

These measures examine the tendency for attention to be drawn more strongly to food stimuli. In this task a food-related picture and a non-food picture appear side by side onscreen for either 500 or 2000 ms, and are then replaced by a dot probe that appears in the position occupied by one of the pictures. The participant has to press the response key corresponding to that position. Attention bias is reflected in faster reaction times for probes replacing food pictures compared with those replacing non-food pictures [50]. GSK1521498 attenuated the attention bias for food stimuli in obese subjects [51] (Fig. 2). However, the D3-antagonist GSK598809 did not affect attention bias to foods except in individuals with low levels of dietary restraint [10].

#### Food related motivation measures

These examine the extent to which an individual is willing to work to attain a food. In a novel grip-force task, participants exerted force on a force transducer to view pictures of food and other rewards. Two pictures were presented at each trial, one clearly visible in the foreground and the other at a very small size in the background. Exerting force on the transducer made the foreground picture recede into the background and vice versa. Motivation was examined in terms of the force exerted to view different images (e.g. the force exerted to see rewarding foods instead of less rewarding foods). At baseline obese subjects exerted greater force to see highly rewarding foods despite no difference in their subjective liking for the foods. However, after 4 weeks on GSK1521498 this effect was no longer present and the liking ratings now discriminated between rewarding and less rewarding foods as would usually be expected [37] (Fig. 2). The force exerted for non-food rewarding images was unchanged, demonstrating a food specific drug effect. Both the above measures could have value in examining the effect of drugs that modulate appetite and satiety, but further work is required to demonstrate their sensitivity to changes in hunger and/or satiety.

#### Laboratory measures of intake and preference

Although monitoring of real world food consumption is difficult and unreliable, being dependent on self report, laboratory measures of taste perception and eating behaviour have all been shown to have good reliability for detecting treatment effects. Taste perception measures examine hedonic responses to varying concentrations of fat and sugar using different dairy composites [52]. As predicted for an MOR antagonist, GSK1521498 significantly attenuated the preference for high fat and high sugar solutions [25].

Measures of intake include buffet meals and *ad libitum* consumption of single item meals (e.g. pasta with tomato sauce). The latter can be combined with a universal eating monitor (UEM), which uses rating measures for hunger and fullness and concealed scales in the serving table to monitor intake, eating rate and meal microstructure. Buffet meals can additionally be used to examine effects on specific food categories and food preference. In obese women sibutramine decreased consumption by almost 16% on an *ad libitum* meal following 7 days of treatment with either 10 mg or 15 mg per day. Greater effects were seen with 15 mg and sibutramine also enhanced within meal satiation [53]. Similar effects were seen with 2 weeks of sibutramine 15 mg (Fig. 2) but importantly, in this study all subjects continued on sibutramine for a further 10 months. Subjects who ate less at the 14 day point lost more weight at 10 months (11.8 ± 6.2 kg; mean ± SD) compared with those who ate more (6.9 ± 2.7 kg) [54]. GSK1521498 reduced caloric intake on an *ad libitum* buffet meal by nearly 400 calories, with a particular effect on the intake of high fat desserts [25] (Fig. 2). These intake measures are simple to administer and would provide valuable information for a potential agent. It is important to emphasise that they can most reliably determine consumption. Food preference is much harder to determine reliably given the multiple factors that determine it, and requires very careful matching of food items for type, nutritional content and flavour across different categories and test sessions.

#### Other potential measures

Decisions about food portions are captured by a measure of expected satiety in which subjects adjust the size of a potential meal serving on a computer to an amount that they think will stave off hunger for a specified duration [55]. Implicit wanting captures food-related motivation in terms of an implicit speeded reaction time when making forced choices between various food pairs [56]. Both these measures are potentially interesting and useful but have not been used so far in studies of dieting or weight loss to allow further comment. When using biomarkers with agents that target appetite and satiety, where measures are taken in the fasted and sated states, it is important to use a standardised satiation manipulation.

## Biomarkers for safety

There has been little work carried out investigating biomarkers for safety. However there are some potentially promising findings that would merit further exploration.

### Functional neuroimaging

Neuroimaging studies of emotion processing examine the neural responses to viewing fearful, happy or neutral faces. In depression there is enhanced amygdala activity in response to fearful faces. This finding improves with even single doses of antidepressants and could serve as a useful biomarker for mood effects [57,58]. GSK1521498 produced no effects on this task (unpublished data). This measure has not been used with rimonabant, and would be important for the newer cannabinoid antagonists and tesofensine, given recent concerns [17] about underreporting of adverse effects (including stress and depression) in the original tesofensine study [16].

### Cognition and behaviour

#### Emotional processing

**Figure 3:**
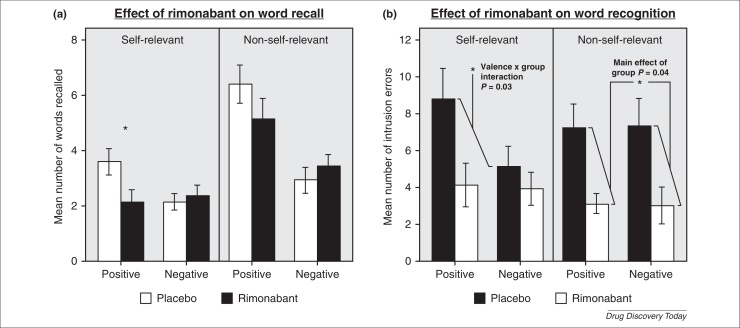
Effect of rimonabant on emotional processing. **(a)** Single dose of rimonabant attenuates the recall of positive self-referent words (personality characteristics) [61]. **(b)** Seven days of treatment with rimonabant attenuates the recognition of positive self-referent words, reflected in greater number of errors in recognising previously encountered words [40]. No effects are seen on negative or non-self-referent words (here animal related words) (*P < 0.05).

Depression is associated with abnormalities of emotional processing including negative bias in the interpretation of ambiguous information, decreased recognition of positive facial emotions and impaired recall of positive self-referent words [59]. These effects have been shown to be manifest early, even before mood is notably depressed and they are improved by single doses of antidepressant treatment [59,60]. In healthy volunteers a single dose of rimonbant 20 mg impaired the recall of positive self-referent words [61], and 7 days of treatment impaired the recognition of previously seen positive words [40] (Fig. 3). In both studies no effects were seen on subjective mood. However, it should be noted that in both studies no effect was seen on facial emotion recognition or other related measures. These findings, although interesting, are nonetheless preliminary and require further exploration.

#### Cognition and sedation

The key point here is the clear need for the use of the standardised cognitive tests for cognition and sedation instead of subjective reports and rating scales. For example, using the power of attention score, which has been shown to be sensitive to drug-induced sedation [62], GSK1521498 was found to have mild sedative effects in Phase I [63]. However, these were found to be mild and transient in Phase II [51]. Rigorous cognitive measures would be important for agents such as zonisamide and topiramate that have documented deleterious effects on memory and cognition. With agents that can have nonspecific effects on reward processing, the specificity of effects can be examined as discussed in the previous section. Finally, although not strictly biomarkers, validated questionnaires and scales for mood, anxiety and suicidality are easily administered and provide important clinically relevant information.

## How would these biomarkers be used in early drug development?

We propose that such biomarkers could be useful in Phase I and II of the drug development process. Simple measures of cognition and tasks such as attention bias can be performed in Phase I alongside safety and dosing assessments. However, it is in Phase II that well designed proof-of-concept studies using such measures could help provide robust signals of proof-of-mechanism or early safety signals. As shown in the previous section, these measures pick up drug effects after short periods of treatment ranging from 1 to 4 weeks, and even after single doses.

One suggested experimental medicine approach in Phase II would be to define the target study population based on the predicted mechanism of action. For example, given the implication of mu-opioid systems in binge eating [64], obese subjects with prominent binge eating could represent a good target population for opioid antagonists. 5-HT2C antagonism is a potential mechanism implicated in weight gain induced by the antipsychotic clozapine [65], and the 5-HT2C agonist lorcaserin might be a good agent to trial in this population (the FDA requires agents for drug-induced weight gain to be trialled in populations taking that specific drug). The agent could then be tested in the sample in a short study of 2–4 weeks using the appropriate biomarkers for the predicted mechanism of action and potential adverse effects. Although it might not be possible to see weight loss with such short durations, biomarker evidence of mechanistic efficacy and safety would support decisions about whether to proceed with further studies. It would be desirable to have biomarkers that could predict future efficacy and even serve as surrogate endpoints but this is more challenging. In the penultimate section we reflect on the challenges and limitations of these biomarkers considering the crucial matter of validation.

## Challenges and limitations

There are several important caveats and challenges to consider with these biomarkers. First, few of these have been used in studies with antiobesity drugs and, in fact, this article has sought to examine all of these. Second, most of these measures and their analysis strategies have not been standardised. To improve their specificity and permit comparisons across different studies and compounds, standardisation is crucial. Third, given the variability seen with these measures across studies, at present most can only reliably be used to track changes from a pre-treatment baseline.

More importantly, there is the crucial matter of validation of these biomarkers. There are two aspects to consider here. The first is the validation of the measure in terms of its performance characteristics and what it measures (i.e. does it reliably and reproducibly measure what it is supposed to in different studies and with different populations?) [26,66,67]. Essential to this, as previously mentioned, is standardisation of the measure and also the formulation of clear measurement endpoints. The biomarker then needs to be consistently replicated in different samples. The second aspect is more challenging and important; can the biomarker predict the clinical endpoint of efficacy and serve as a surrogate endpoint? To determine this requires prospective larger scale and longer term studies that can be used to model how the biomarker and the process it captures predicts the clinical endpoint [26,66,67].

Surrogate endpoints will be a considerable body of work and it can be argued that surrogate endpoints can have limited value in antiobesity drug development given they are very unlikely to support regulatory approval. One approach might be to apply these biomarkers to the study of existing compounds in short studies in healthy obese individuals [28,68,69]. A more pragmatic approach might be to include some of these measures in the current design of Phase I, II and even Phase III studies with new compounds to build up an evidence base [11,37]. There is of course the matter of cost: measures such as fMRI, particularly using the necessary repeated measures, are expensive. However the cost of running well-designed short proof-of-concept studies would be far less than that of large Phase II and III trials.

The more pressing question is: what does a biomarker add to a measure of weight change? It is a reasonable contention that, under current regulations, there is no reason to advance a potential agent in the absence of a weight signal. Conversely, if an agent does produce significant weight loss it can be argued that biomarkers for proof-of-mechanism are not required and safety concerns can arguably be reasonably determined in Phase II and III. We would contend that weight loss without clear proof-of-mechanism would be an unsatisfactory, although clearly not untenable, position for a centrally acting agent. If there is proof-of-mechanism with weight loss, biomarkers can facilitate dose optimisation and the formulation of potential combination treatments. What if there is proof-of-mechanism but no weight loss? If dosing has been optimised, this might suggest that the mechanism targeted is insufficient to affect weight loss alone but could be potentially valuable as part of a combination treatment with other pharmacological agents or with targeted behavioural interventions, or as a potential agent for preventing weight regain. Although the FDA and EMEA expect that a drug should demonstrate efficacy for weight maintenance after weight loss, given the current pharmacopoeia and regulatory framework it could be some time before the idea of an agent purely for weight maintenance gathers any traction, if at all. Finally, it is important to note the value that these measures have in the characterisation of the neural systems of appetite and reward in humans. This latter point is key because it is only through a sophisticated understanding of the pathways towards obesity that therapies can be optimised and targeted effectively.

## Concluding remarks

We have presented a case for the potential value of CNS biomarkers in antiobesity drug development, and identified some promising candidates that have been used thus far. However, the field is still in its infancy and there remains a great deal of work to be done before these biomarkers can be standardised and validated and even more if they are to be used as potential surrogate endpoints. We accept that in the current regulatory requirement their value might not be immediately apparent but they could become invaluable as we work towards more sophisticated conceptualisations of obesity and antiobesity treatments.

## Conflicts of interest

During the preparation of this manuscript H.Z. was a Translational Medicine and Therapeutics (TMAT) Clinical fellow jointly funded by the Wellcome Trust and GlaxoSmithKline (GSK). H.Z. and P.C.F. have worked with GSK on some of the studies cited in this manuscript, and are involved in ongoing collaborations with GSK.

*Chemical compounds studied*: Rimonabant (CID 104850); sibutramine (CID 5210); GSK1521498 (CID 71301379); lorcaserin (CID 11673085); Qsymia^®^ (CID 56842108); tesofensine (CID 11370864); velneperit (CID 20629114); liraglutide (CID 44147092).

## Figures and Tables

**Table 1:** Potential biomarkers for antiobesity drug development

**Examining mechanisms of action**			
**Biomarker**	**Mechanism**	**Application with antiobesity drugs**	**Potential future study candidates**
*Neuroimaging (fMRI)*			
Food pictures task Examination of neural responses in reward and appetite control regions: ventral striatum, orbitofrontal cortex, insula, amygdala, putamen, hypothalamus	Attenuation of appetitive drive/enhanced satiety	Sibutramine (+) [23]	RM403 (+), Lorcaserin (+) Liraglutide (+), Velneperit (+), Tesofensine
	Attenuation of food related reward	Rimonabant (+) [35]	Neutral cannabinoid antagonists (−)
	Attenuation of food specific reward	GSK1521498 (+) [32] GSK598809 (−) [7]	Neutral cannabinoid antagonists (−)
*Cognitive and behavioural*			
*Ad libitum* food intake Intake on buffet meals, or *ad libitum* consumption on single meals with or without universal eating monitor (UEM)	Attenuation of appetitive drive/enhanced satiety	Sibutramine (+) [47,48] GSK1521498 (+) [20]	All above
Attention bias to food pictures Speeded reaction time when target occurs in location closer to food cue	Attenuation of salience of food cues	GSK1521498 (+) [45] GSK598809 (−) [6]	Tesofensine
Motivation for food rewards: picture surfing task Greater force exerted to view rewarding foods	Attenuation of motivation for food rewards	GSK1521498 (+) [32]	Tesofensine
Expected satiety and satiation Estimated portion size that will stave off hunger or induce sufficient fullness	Attenuation of appetitive drive/enhanced satiety	Not used so far	Requires testing Drugs that enhance satiety/decrease appetite
Implicit wanting Speeded reaction time in forced choice comparisons of different food types	Attenuation of motivation for food rewards	Not used so far	As above

**Examining potential neuropsychiatric adverse effects**			
**Biomarker**	**Mechanism/adverse effect**	**Application with antiobesity drugs**	**Potential future study candidates**
*Neuroimaging (fMRI)*			
Neural responses to emotional face processing in amygdala: enhanced amygdala responsiveness to fearful faces	Mood, anxiety	GSK1521498 (−) unpublished data	Neutral cannabinoid antagonists (−)
*Cognitive and behavioural*			
Emotional processing: Decrease in positive bias for self-relevant words on recall or recognition	Mood, anxiety	Rimonabant (+) [55,56]	Neutral cannabinoid antagonists (−)
Emotional face processing Enhanced recognition for fearful faces	Mood disturbance	Rimonabant (−) [55,56]	Neutral cannabinoid antagonists (−)
Measures of cognitive speed: For example power of attention score	Sedation	GSK1521498 (−) [45]	Empatic (−) Contrave (−)
Measures of memory: For example CANTAB	Memory disturbance	Not used so far	Empatic (−) Contrave (−)

In the third column (+) indicates that an effect was seen and (−) indicates that no effect was seen. In the fourth column the symbols indicate the desired effect for future agents.
